# The digital age

**DOI:** 10.1080/16549716.2017.1344030

**Published:** 2017-08-25

**Authors:** Carl Bildt

**Affiliations:** ^a^ Prime Minister of Sweden (1991-1994)

Across the globe, policymakers are starting to understand that we are on the verge of major transformations in virtually all areas as the different digital technologies continue to develop.

**Figure:**
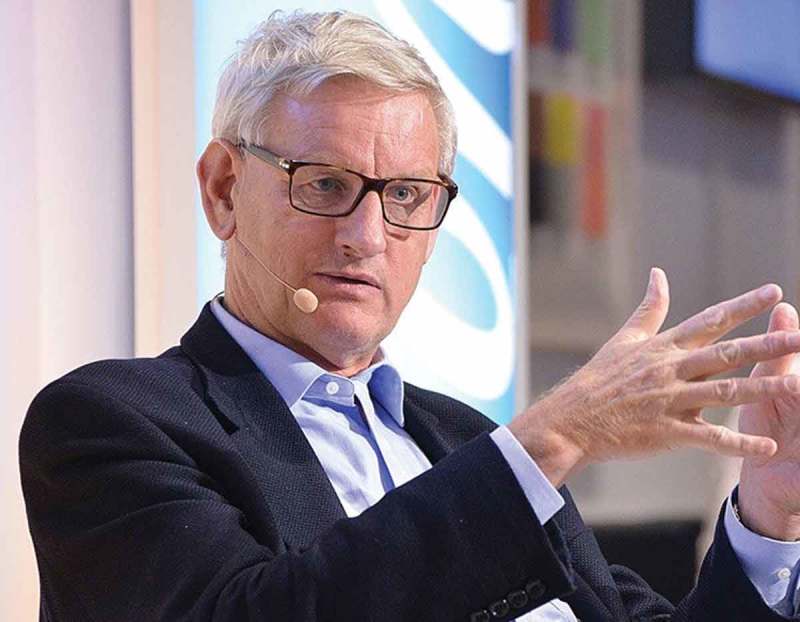

The catchwords are there everywhere.

There is talk of the coming Internet of Things. In China what is called Internet Plus is at the centre of long-term planning. India is advancing its Internet India plan for rapidly expanding connectivity. Germany is talking about Industry 4.0, and the EU is working on creating a Digital Single Market.

We are in the beginning of the digital age.

Mobile communications are now taking centre stage in this development, and, in particular for countries that were not in the forefront of the Industrial Revolution of previous centuries, this will be key to their opportunities.

On present trends we will see 90% of the population of the world within half a decade or so being covered by mobile broadband networks with a capacity equal to or better than the European average today. And while there is concern over the geographical digital divide when it comes to actually accessing the networks, I think the generational digital divide will be more pronounced. With prices rapidly coming down, most young people all over the world not too far into the future will have some sort of smart device connecting them to the Net.

This will happen in parallel with the gradual introduction of the 5G networks which will provide radically improved capacity. We are talking about improvements by a factor of a hundred in some important respects. And this will also open up the possibility for numerous new areas of application.

The health sector is clearly one of them. From fast and easy access to basic health information to the possibility of remote diagnostics and even surgery, the provision of health services, not least in developing countries, could also be transformed.

For this to be possible, it is of course imperative that access is there at prices that are affordable, and here it is important that the lessons of the past few decades are properly understood.

Sweden took an early lead in the development of the Net economy as well as mobile communications. The fact that we had a domestic industry was not the decisive factor, but rather that we created a very open and highly competitive framework for the different actors to operate in. This stimulated investment, drove down prices and led to a rapidly increasing demand for services.

Other countries provide examples of less successful practices. Efforts to protect incumbent – sometimes public, sometimes just well-connected – operators have invariably led to higher prices and a slower development of the overall market.

That does not mean that there is no role for the public sector and for the state. An open and transparent market does require open and effective regulation. And the public sector also has a key role in demanding and demonstrating the application of digital services in all its different activities. It can be a vanguard in the digital transformation.

During recent years, increased attention has been given to different aspects of cyber security, and, as digital functions are becoming increasingly important to the functioning of our societies these are very important concerns. Here, as well, the public sector has an important role to perform.

Of fundamental importance is to secure as high a standard of what we might call digital hygiene as possible. It is when basic issues such as password management are not handled properly that there are openings for viruses, hackers and other non-benevolent actors to enter the networks and create damage. Responsibility for cyber security rests with each and everyone accessing the networks.

In terms of use of these technologies, issues related to the integrity of the data become particularly important. It can have fatal consequences if data is tampered with or is degraded in some way. Here the emerging global debate on the use of encryption is of particular importance. Much as it is important to protect the security of the networks we are so dependent on, it is even more important to protect the integrity of the data [1].

We are only in the beginning of the digital revolution. A decade from now, virtually everything will be different with much improved connectivity, vastly improved applications, more experience of use in providing different public services and, hopefully, more attention given to the essential cyber security issues.

But it is now that it is imperative for policymakers in different areas to start to focus on all of the issues associated with this development.
